# Rotation-independent representations for haptic movements

**DOI:** 10.1038/srep02595

**Published:** 2013-09-05

**Authors:** Satoshi Shioiri, Takanori Yamazaki, Kazumichi Matsumiya, Ichiro Kuriki

**Affiliations:** 1 Research Institute of Electrical Communication Tohoku University

## Abstract

The existence of a common mechanism for visual and haptic representations has been reported in object perception. In contrast, representations of movements might be more specific to modalities. Referring to the vertical axis is natural for visual representations whereas a fixed reference axis might be inappropriate for haptic movements and thus also inappropriate for its representations in the brain. The present study found that visual and haptic movement representations are processed independently. A psychophysical experiment examining mental rotation revealed the well-known effect of rotation angle for visual representations whereas no such effect was found for haptic representations. We also found no interference between processes for visual and haptic movements in an experiment where different stimuli were presented simultaneously through visual and haptic modalities. These results strongly suggest that (1) there are separate representations of visual and haptic movements, and (2) the haptic process has a rotation-independent representation.

We move our hands and arms to write, draw, and gesture, referring to representations of actions and/or images in memory, which can be obtained through active/passive haptic movements or through visual information on another person's movements. Both visual and haptic inputs can ultimately be used for haptic movements. Yet to be elucidated, however, is whether visual and haptic information are represented in the same system. Although interaction between the inputs is known for motor skill acquisition^1^, it does not answer the question of what are the representation processes. Different representations for haptic movements from visual ones likely have an advantage in body movement control without the restriction of a coordinate system fixed to the visual system: The visual system usually uses a coordinate system referring to the vertical axis as the normal, while the motor process has its own coordinate system for movement control as suggested by the difference in subjective vertical between vision and haptics^2^,^3^. The representation of haptic movements might have fewer constraints in spatial coordinates to allow greater freedom for control. Note that here we consider passive haptic movements and another person's movements as inputs, although our interest is in active haptic movements. This is because the motor cortex is activated by passive movements^4^,^5^ and similar brain activity is found for action and visual motion perception^6^,^7^.

Several psychophysical studies have found that signals from visual and haptic modalities are processed in a common multimodal representation system^8^,^9^,^10^,^11^,^12^,^13^,^14^,^15^,^16^,^17^,^18^. Several functional magnetic resonance imaging (fMRI) studies have also shown involvement of common brain areas in visual and haptic recognition^19^,^20^,^21^,^22^,^23^, which also suggests a common process for visual and haptic representations. Representations of haptic movements, however, might be processed in a system different from the one for object representations. For the visual process, there is a known dichotomy between the motion/space/action-related (dorsal) pathway and the color/object-related (ventral) pathway^24^,^25^,^26^,^27^. There might also be different haptic processes for object and movement information. Knowledge of the object representation process might not help to understand the representation of movement signals obtained through haptic perception.

Because actions are often based on visual information, representations of haptic movements and their relationship with vision should be as important as or even more important than representations for objects, particularly when one mimics another person's limb movements, as suggested by studies on the mirror-neuron system^28^: A mirror neuron responds to both one's own action and observation of the same action performed by another. To transform someone's action into self-action, haptic representations independent of visual representations potentially play an important role because visual representations are usually related to physical space/objects and are often viewpoint-dependent.

Despite this importance, no study, to the best of our knowledge, has examined whether a modality-specific or multimodal process contributes to movement representations. On the one hand, a few studies have suggested a common process for visual and haptic motion perception^29^,^30^, but that is about perception of object motion, not haptic movements. On the other hand, hand movements have often been used for object perception^22^,^31^, but our interest is in representation of movements themselves, such as those of drawing characters. We compared the characteristics of an imagery task involving mental rotation^32^,^33^,^34^,^35^ between visual and haptic movements using a virtual display of both visual and haptic information (Fig. 1). Although similar effects of rotation angle have been reported for visual and haptic object representations^8^,^36^,^37^, and the contribution of parietal and premotor cortices to mental rotation of visual stimuli has been suggested^38^, investigation of movement representations is a different issue. No study has compared the effect of rotation angle on reproducing movement patterns obtained through visual and haptic inputs, although stimulus objects have often been explored with haptic movements^22^,^31^,^39^. Perhaps, we obtain a mental rotation effect for visual stimuli similar to the effect obtained with static visual images because a movement pattern can be easily represented as a static line drawing. In contrast, we might not obtain a similar effect for haptic stimuli because haptic information without reference to a particular axis is possibly advantageous for controlling limb movements. This contrasts with object recognition, where haptic and visual representations have the benefit of sharing the same coordinate system independent of source modality.

For mental rotation stimuli, we used two-stroke patterns that were expressed by movements of a visual stimulus or a passive haptic movement. A computer controlled the movements in both cases. There was a learning phase and a test phase (learning indicates temporal memorization of movement patterns, not training of a motor skill). In the learning phase, a movement pattern was presented to the participant either visually on a display or haptically via a force-feedback device. To present visual patterns, a computer moved a yellow disk on the display. To present haptic patterns, a computer moved the stylus of a force-feedback device held in the participant's right hand. The stylus pulled the participant's hand, and the participant perceived the movement pattern through the passive hand movement. The stylus was static in the visual presentation, and the display was dark in the haptic presentation.

In the subsequent test phase, the pattern was rotated in an angle, and the first stroke was presented. The participant's task was to recall the learned pattern and to show the second stroke (Fig. 1b). There were visual and haptic test conditions. A computer presented the first stroke by the movement of the yellow disk in the visual test. The participant was instructed to indicate the end point of the second stroke by the cursor (the same yellow disk), which was moved with the force-feedback device (Fig. 1c). In the haptic test, the computer presented the first stroke by the stylus's movement, and the participant was instructed to draw the second stroke by moving the stylus (no force-feedback except for the reaction force from the virtual plane). Although the participant used the stylus in both visual and haptic test conditions, the task in the visual test was to place the visual cursor at the terminal point of the imaginary second stroke, and that in the haptic test was to move the stylus to draw the second stroke without visual feedback. Because the first stroke was given visually and because the participant was instructed to move the visual cursor on the display, the visual signal was assumed to be used dominantly in the visual test. Similarly, because the first stroke was given haptically without visual stimulation and because the participant was instructed to draw the second stroke, the haptic signal had to be used in the haptic test. In both test conditions, we measured latency to start the stylus's movement. This is a mental rotation task because the participant had to rotate the representation of the learned pattern to perform the task, although this differs from conventional normal/mirror image discrimination^32^,^33^,^34^.

To isolate differences in representation between visual and haptic processes from other response-related effects, we used four combinations of learning and test modalities: visual learning and visual test (VV), visual learning and haptic test (VH), haptic learning and visual test (HV), and haptic learning and haptic test (HH). Common features between VV and VH reflect characteristics of visual representations, and those between HV and HH reflect characteristics of haptic representations.

## Results

We found different effects of stimulus rotation between visual and haptic learning conditions independent of test conditions. The average response latency, which is the average of the individual median latencies, increased with rotation angle for the visually learned stimulus. In contrast, we observed a much weaker effect of rotation angle for the haptically learned stimulus (Fig. 2). A conventional mental rotation effect, longer time for larger rotations^32^,^33^,^34^,^35^, was found in the visual learning condition with the haptic test as with the visual test. In contrast, latency showed a difference between only the 0° and 90° rotations for haptic learning, also in both types of tests. These findings are confirmed by statistical tests. A three-way within-subjects analysis of variance (ANOVA) (learning modality, test modality, and rotation angle) showed significant main effects of test modality (F(1,7) = 532.99, p < 0.0001) and rotation angle (F(3,21) = 14.09, p < 0.0001), and interactions between learning modality and rotation angle (F(3,21) = 3.08, p < 0.05). We also tested the effect of order, separating data in the first and second sessions. A four-way ANOVA (first/second sessions, learning modality, test modality, and rotation angle) showed significant interactions between learning modality and rotation angle (F(3,21) = 4.32, p < 0.01), whereas no significant interaction was shown among learning modality and rotation angle and first/second sessions (F(3,21) = 0.09, p > 0.1). We also found shortening of latency (approximately 4% on average) in the second session (F(3,21) = 14.2, p < 0.001) as expected from practice and/or learning effects.

To test statistical differences among rotation angles following the ANOVA results, we performed multiple comparisons between all combinations of rotation angles, except between 90° and 270° (or −90°), where the angle difference from the original angle was the same. We used averages of the visual and haptic test conditions for the test because the ANOVA showed a significant interaction between rotation angle and learning modality, but not between test modality and rotation angle. With Holm's correction, a *t*-test showed significant differences between all five comparisons for visual learning. The same test showed a significant difference between only 0° and 90°, 180°, or 270° for haptic learning (p < 0.05).

There was a trivial effect in response accuracy between rotation angles, as in previous visual mental rotation experiments^32^,^34^. Although the ANOVA showed a significant main effect of rotation angle (F(3,21) = 8.65, p < 0.0001), no interaction was found between any two factors. The effect was very small, and less than 15% of the standard deviation (Supplementary Fig. 1). The *t*-test with Holm's correction showed significant differences only between 0° and 90° or 270° for the data averaged over all learning and test conditions (p < 0.05). It is not surprising that there was a benefit in task performance in the 0° condition, where the test stimulus was a repetition of the learned stimulus.

These results are consistent with the hypothesis that there are different representation processes between visual and haptic information. Haptic movements are coded as rotation-independent representations as opposed to visual representations that depend on rotation angle. It should be noted that we do not expect an interaction between test modality and rotation angle here. If visual and haptic information is stored through separate mechanisms, the effect of test angle should be the same for a given learning modality, but does not have to be the same for a given test modality. Although more time might be required to access the representation when different modalities are used between learning and test phases, no difference in the effect of rotation angle is expected with the same learning modality as far as mental rotation of the memorized representation is concerned. This is not only a strong piece of evidence for the separate representations of vision and haptic movements, but also a finding of a unique feature of the haptic movement process. This, however, is not to say that visual imagery is never obtained through haptic stimulation. Even if the participants constructed visual representations more or less from haptic movements, these results suggest that the visual representation obtained from haptic learning was not used, at least not as often as the haptic representation.

The difference found between 0° and other angles for haptic learning appears to complicate the interpretation of the results. Such a difference is not consistent with the assumption of rotation-independent representations. The difference can be attributed to a specific effect of repeated movements in the condition with 0° rotation. In the HH condition with 0° rotation, haptic movement was identical between learning and test phases. Identical movements could lead to further decreases in latency in the second movement. To examine the possible effect of repeated movements, we performed additional experiments using HV and HH conditions. The only difference from the main experiment was an interposed task between the learning and test phases. The participant was asked to track a circularly moving target on the display by using a visual cursor controlled by the haptic device for 4 s between the two phases. Latency data showed no difference between 0° and 90° rotations and no difference between rotation angles in either condition (Fig. 3). The same ANOVA as in the main experiment showed a significant effect for only the test condition (F(1,7) = 236.20, p < 0.0001). The difference between 0° and 90° rotations for haptic learning in the main experiment can be attributed to a facilitation effect due to repetitive arm movements between learning and test phases.

The existence of two independent processes for visual and haptic representations raises an important question. Are visual and haptic representations integrated as a multimodal representation? Differences between learning modalities independent of test modalities suggest no integration of visual and haptic representations. If there is a common process, the process is likely used for efficient task excursion when different modalities are used in the learning and test phases. To directly address this issue, we conducted a second experiment. In the second experiment, we used simultaneous presentations of visual and haptic movements to investigate interference between the two types of representations. We would expect no interference if visual and haptic representations were never integrated. After simultaneously presenting visual and haptic movements in the learning phase, we presented an auditory cue in the interval between the learning and test phases to inform which of the visual or haptic movements to recall (one beep for the visual stimulus and two beeps for the haptic stimulus). The participant thus had to memorize both visual and haptic movements for the task in the test phase. There were consistent and inconsistent trials. Visual and haptic movements were the same in consistent trials and different in inconsistent trials (Fig. 4).

Results in the inconsistent condition revealed little or no interaction between visual and haptic learning/memory processes (Fig. 5(A)). Even when visual and haptic information is memorized simultaneously, latency is similar to that under single modality conditions. Moreover, the effect of rotation angle is specific to the memorizing modality, as under single modality conditions.

A four-way within-subjects ANOVA (learning modality, test modality, consistent/inconsistent, and rotation angle) showed significant main effects of test modality (F(1,7) = 569.61, p < 0.0001) and rotation angle (F(3,21) = 11.31, p < 0.0001). We found a significant interaction between rotation angle and learning modality (F(3,21) = 3.86, p < 0.05) as in Experiment 1. A three-way interaction among consistent/inconsistent, test modality and rotation angle was also significant (F(3,21) = 3.89, p < 0.05). In inconsistent trials, the *t*-test showed significant differences (p < 0.05) between all five comparisons for visual learning, whereas it showed a significant difference (p < 0.05) between only 0° and 90°, 180°, or 270° for haptic learning with Holm's correction.

Although we had no particular prediction for consistent trials, we expected some kind of mixture of two representations in response because both visual and haptic representations were available in the test. The general trend of the results of the consistent trials is similar to that of visual learning in Experiment 1 (Fig. 5(B)). However, latency functions are somehow between the visual and haptic learning results in Experiment 1. Rotation angle clearly changed latency, but the amount of the angle effect was smaller than that of visual learning in Experiment 1 and that of the inconsistent trials, consistent with the significant three-way interaction among consistent/inconsistent, test modality, and rotation angle. The *t*-test showed significant differences (p < 0.05) between all five comparisons for haptic learning and all but one comparison for visual learning (except for that between 180° and 270°). This appears to indicate interference between the two representation systems, which disagrees with the concept of independent representations for visual and haptic movements. However, this pattern of results can be predicted by assuming random selection from two independent representations (see next section).

### Probabilistic interaction of visual and haptic representations

We investigated whether independent visual and haptic representations are sufficient to predict the apparent interference shown by consistent trials in the second experiment. In a consistent trial, the participant should use either one of two representations for performing the task under the assumption that two processes work independently. The participant can choose either representation randomly from trial to trial with a certain probability for each choice. In such a case, median latency can be determined from a mixture of latency distributions for the visual and haptic representations.

We calculated latency results in consistent trials by using a model of random selection with latency distributions of inconsistent trials. Assuming that each of the two representations was chosen with a certain probability (α and 1 − α for visual and haptic representations, respectively, and α varied with an interval of 0.05), we obtained a latency distribution of consistent trials for each participant by using 2100 random samples (the number was set to be a multiple of 21, because 1/21 was the step of probability changes). Then, we calculated median latency from the distribution of each individual to obtain an average over individual medians. We repeated the procedure 1000 times and obtained the average for comparison with the actual results (Supplementary Fig. 2). By comparing the prediction error with variable α values, we chose the α with the minimum least square error for each of the visual and haptic responses. With the best α value, the random selection model predicts experimental data well, both in terms of shape and absolute values (Fig. 6): The value of α is respectively 0.6 and 0.75 for visual and haptic response conditions. These values indicate a greater contribution of visual representation than haptic representation, independent of response type.

Integration of different signals in relation to Bayesian theory has been investigated in several cue integration studies^40^,^41^,^42^. Ernst and Banks, for example, showed that visual and haptic signals are averaged with weights determined by signal reliability^40^. We examined whether the same rule applied to the relative contribution of visual and haptic representations in consistent trials. Assuming independent visual and haptic processes, our analysis showed a relative contribution, or probability of selection, of about 0.7 (0.6 and 0.75 for the two response conditions) for visual representations and 0.3 for haptic representations. The larger relative contribution of visual signals might be related to the higher reliability of visual representations relative to that of haptic representations. Reliabilities of the signals can be estimated from the precision of the response, that is, the standard deviation of goal direction indicated by participants. Standard deviations in angle variation for the responses were 49.6°, 56.7°, 57.1°, and 57.8° for VV, VH, HH, and HV, respectively. Reliability was slightly higher (standard deviation was smaller) for the visual learning condition than for the haptic learning condition with visual response, whereas little difference was seen for haptic response. Relative contributions for visual response were 0.55 for visual learning and 0.45 for haptic learning, and those for haptic response were 0.50 and 0.50. The signal selection ratio that we estimated for our latency results (0.60 vs. 0.40 or 0.75 vs. 0.25) was not likely directly related to signal reliability. Signal selection might be fundamentally different from signal integration.

## Discussion

Representations of haptic information do not seem to have a particular angle of reference and thus can be considered rotation-independent representations. This study indicated that no cost is required to use haptic movement representations with an angle different from a memorized one. This contrasts with visual representation, which requires longer time to rotate larger angles. The difference might be related to the reference axis in the representation system. There is perhaps no physical direction that is special for haptic movements, whereas the vertical direction is special for visual perception. One can draw a letter similarly on a front parallel plane or on a plane at either side of the body. Haptic information has the benefit of being represented relative to the body. However, the rotation-independent representation we found is a more general property of haptic perception rather than a property specific to the motor control system. A hand-centered representation cannot explain the results of the present experiment because the haptic device was held and the hand position was similar for all rotations in the experiments. If haptic movements are represented, for example, in the shoulder coordinates, there is no reason to expect the absence of the mental rotation effect. We suggest that the difference between visual and haptic representations for movements is not simply due to the coordinate system, but due to a qualitative difference in represented information. Such a representation might be related to the mirror-neuron system. When one tries to mimic another person's movements as visually observed, the movement information observed and that used for self-movements are represented from different viewpoints: the third- and first-person viewpoints. The system with rotation-independent representations can be used to transfer one from the other and/or to integrate them. The method developed in this study has the potential to reveal this relationship in future studies.

The rotation-independent representation contrasts with the representation process for haptically perceived objects. Mental rotation effect has been reported with haptic objects^8^,^36^,^37^ and mutual interference between haptic and visual stimulation has also been reported^22^,^43^. These studies suggest a common process for haptic and visual object representations. However, the representation for objects and that for movement signals are possibly very different. Indeed, they are processed in different pathways for vision^24^,^25^: object recognition in the ventral pathway, and movement perception in the dorsal pathways. For haptic signals, there are suggestions that both dorsal and ventral pathways contribute to haptic perception but in different ways^20^,^21^,^39^. The dorsal pathway likely processes haptic texture and the ventral pathway likely processes haptic objects^20^,^21^. Moreover, representations for texture are perhaps rotation independent because no particular reference axis is required for texture perception. Our finding of the rotation-independence in haptic movement representation, which differs from that in haptic object representation, is consistent with the dichotomy of haptic processes.

## Methods

### Experiment 1

#### Stimulus

**Figure 1:**
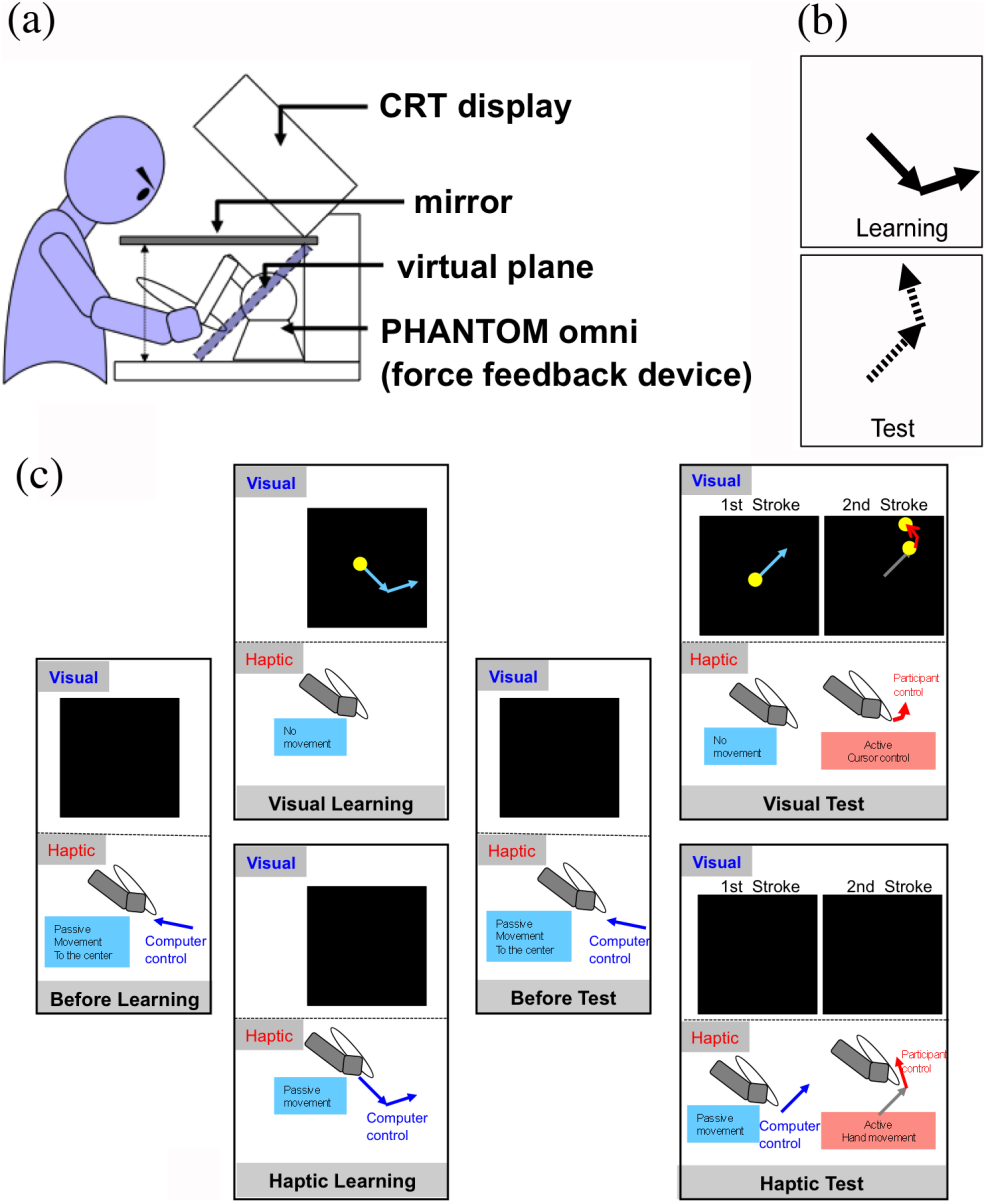
(a) Experimental setup. Visual stimuli were presented on a cathode ray tube display, which the participant viewed through a mirror, behind which the participant moved the stylus of a force-feedback device with the right hand. There was a virtual haptic plane corresponded to the virtual visual display. (b) The stimulus was a movement pattern of two strokes. (c) Visual and haptic stimulation in each phase of each modality. In visual learning, a computer moved a yellow disk on the display. In haptic learning, the computer moved the stylus of a force-feedback device held in the participant's right hand. In the visual test, the computer moved a yellow disk to display the first stroke, and the participant moved a cursor (the moving disk for the first stroke) to the end point of the second stroke by moving the stylus. In the haptic test, the computer moved a stylus to display the first stroke pulling the participant's hand, and the participant drew the second stroke while continuously moving the stylus. Before each learning and test phase, the stylus and hand were moved to the central location.

**Figure 2:**
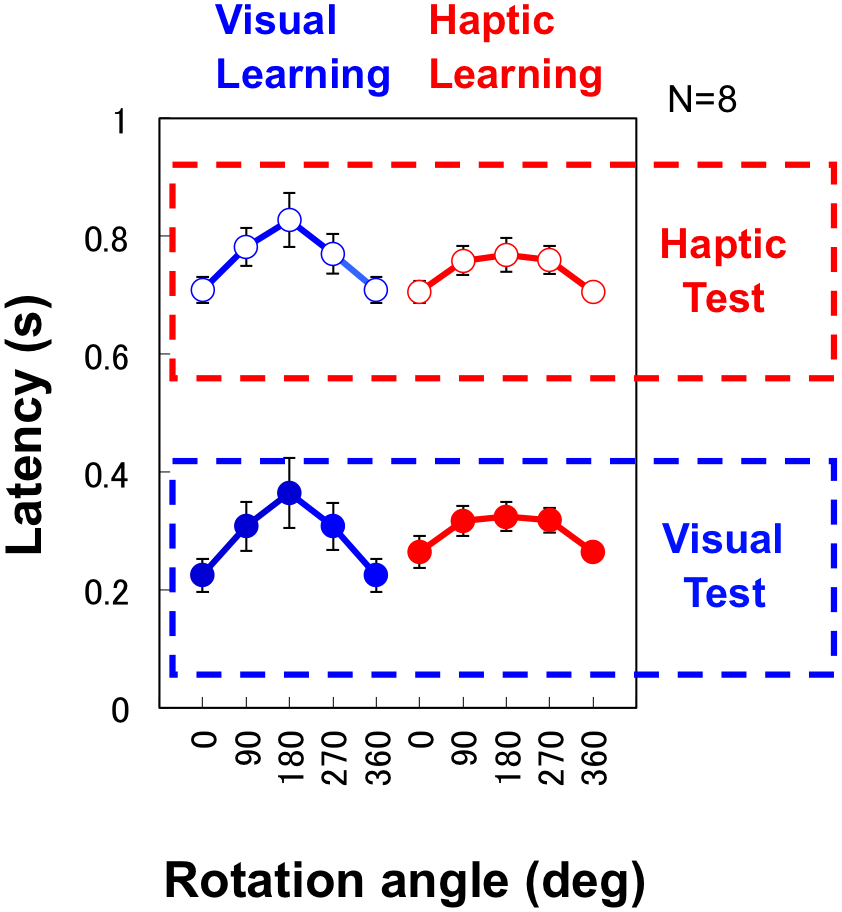
Average of median response latencies of all participants plotted against rotation angle. Response latency is longer for larger rotation angles (the largest rotation angle is 180°) for visual learning, whereas a clear difference is seen only between 0° and other rotations for haptic learning. Each function shows latency data for one of the four combinations of visual/haptic learning/test conditions. Error bars indicate standard error of the mean across participants, and the plot at 360° is a replica of that at 0°.

**Figure 3:**
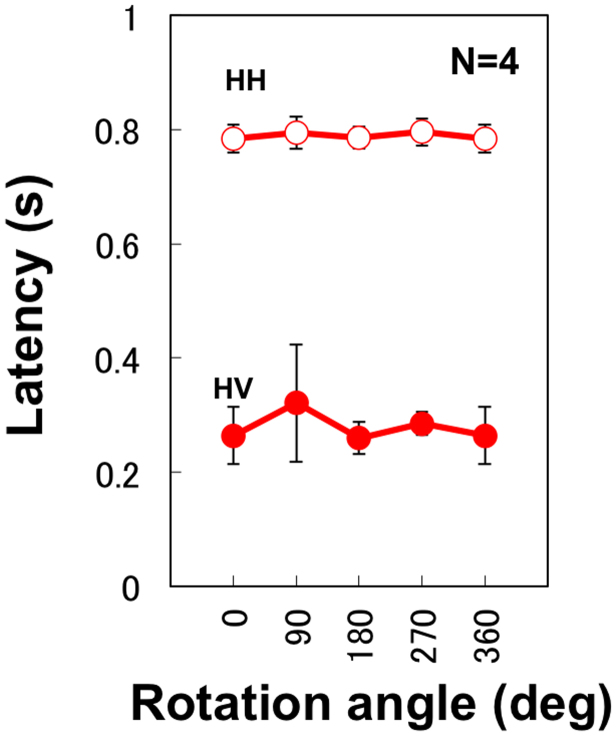
Response latency plotted against rotation angle for the control experiment with an interference task between learning and test phases. The difference between 0° and other rotations for haptic learning found in the main experiment disappeared. Error bars indicate standard error of the mean across participants, and the plot at 360° is a replica of that at 0°.

The stimuli were two stroke patterns (Fig. 1). The length of each line segment was between 40 and 50 mm (6.0° and 7.5° in visual angle), and the angle between the two segments varied randomly. The visual stimulus was a yellow disk with a diameter of 4 mm (0.6° in visual angle). Disk luminance was 125 cd/m^2^ with CIE1931 *xy*-coordinates (0.40, 0.50) against a black background (0.75 cd/m^2^). The haptic stimulus was movement of the stylus of a force-feedback device. A computer controlled the force needed to pull the stylus on a virtual plane. Movement speed of the disk and stylus was 6.0 cm/s (9.0°/s).

#### Apparatus

Visual stimulus was presented on a cathode ray tube display (Iiyama, HF703U), and the participant viewed it through a mirror, behind which the participant moved the stylus of a force-feedback device with the right hand (Fig. 1). The participant felt as if drawing with a pen when the locus of the cursor movement was shown (locus was never shown during the experiment). The left hand was relaxed on the desk or knee. Display size was 32.5 × 24.5 cm and the refresh rate was 75 Hz. Viewing distance was 38 cm. The force-feedback device (PHANTOM Omni, Sensable) was positioned so that the point of the stylus and the cursor on the display coincided. A virtual haptic plane corresponded to a virtual visual display behind the mirror. Position data were obtained from the force-feedback device at the same rate as the display's refresh rate (75 Hz). Experiments were performed in a room without any light source other than the display.

#### Procedure

We used four combinations of haptic and visual stimuli for the learning and test phases. We used either visual or haptic stimulus in the learning and test phases: visual learning and visual test (VV), visual learning and haptic test (VH), haptic learning and visual test (HV), and haptic learning and haptic test (HH). In the learning phase, a two-stroke stimulus was presented either by a moving visual disk or by a haptic force on the participant's hand to draw lines.

The direction of the first stroke in the learning phase was randomly chosen from 45°, 135°, 225°, and 315° in each trial, and the angle between the two lines was also randomly chosen in each trial, with the restriction that the same percentage of trials be selected for eight evenly divided ranges of angles: −22.5° to 22.5°, 22.5° to 67.5°, …, 292.5° to 337.5°. In the test phase, the first stroke was given after the stimulus was rotated by 0°, 90°, 180°, or 270°. The task was to recall and indicate the second stroke as soon as possible after the computer presented the first stroke, mentally rotating the learned stimulus similarly to a conventional visual mental rotation task (although the task might be performed without mental rotation in haptic learning).

A trial started when the participant pressed a button on the haptic device while fixating on a fixation spot on the display. The location of the fixation spot, which corresponded to the stylus location, was randomly determined within a central 20 mm square area at the center of the display. The haptic device pulled the participant's hand by means of the stylus to the location. After a blank interval chosen randomly between 2 and 3 s, a learning stimulus was presented either visually or haptically. Before the test phase, a blank interval was also randomly chosen between 2.5 and 3.5 s. During the interval, the haptic device pulled the participant's hand again to the central area with the same positional randomization. The participant's task was to recall the learned pattern and to show the second stroke in an appropriate rotation. The computer presented the first stroke by movement of the yellow disk in the visual test. The participant was instructed to indicate the end point of the second stroke by the cursor on the display, which was moved by the force-feedback device. In the haptic test, the computer presented the first stroke by the stylus's movement, and the participant was instructed to draw the second stroke by moving the stylus from the end location of the first stroke.

Each session consisted of 128 trials (4 rotations × 4 first-line directions in the learning phase × 8 ranges of the angle between the strokes), and each participant performed two sessions in each of the four conditions. For all participants, conditions were run in a fixed order of VV, VM, MV, and MM in the first session and in the reverse order in the second session.

#### Participants

Eight students at Tohoku University participated in the experiments (age range 22–24 years). All had normal or corrected-to-normal visual acuity. All participants were right-handed and held the stylus with their right hand. They had experience in psychophysical experiments but did not know the purpose of the experiment. The research was conducted according to the principles expressed in the Declaration of Helsinki. The experiments were undertaken with the understanding and written consent of each participant.

### Experiment 2

#### Procedure

The stimulus, apparatus, and participants were the same as in Experiment 1. The experimental paradigm was similar to that in Experiment 1 except that visual and haptic movements were presented simultaneously in the learning phase. Either a visual or haptic stimulus was used in the test phase of a session. Because which of the visual or haptic pattern was presented in the test was informed by an auditory cue in the interval between the learning and test phases (one beep for visual and two beeps for haptic), the participant had to memorize both visual and haptic movements to perform the task appropriately.

**Figure 4:**
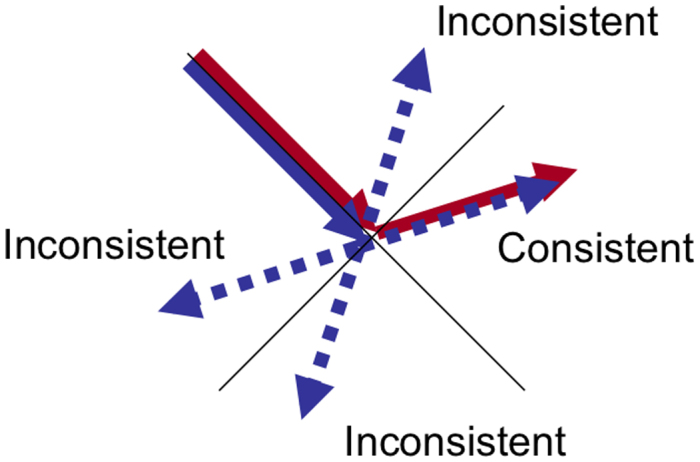
Visual and haptic movements in consistent and inconsistent trials. To realize similar conditions between consistent and inconsistent trials, we used inconsistent movements symmetrical along either the axis of first stroke or the perpendicular axis. Solid arrows indicate consistent trials and dashed arrows indicate inconsistent trials.

**Figure 5:**
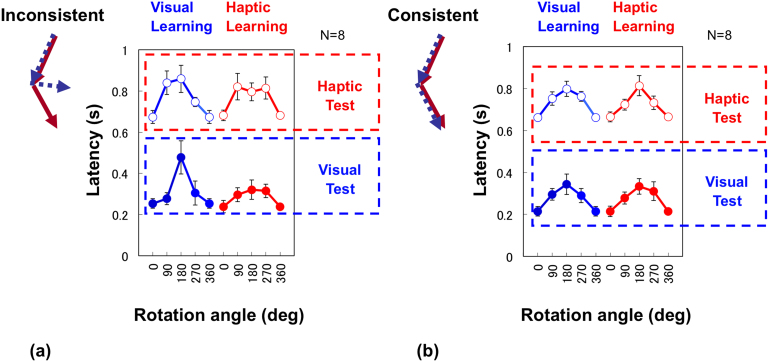
Response latency plotted against rotation angle for inconsistent trials (a) and consistent trials (b) Effect of rotation angle for inconsistent trials is similar to that in Experiment 1, suggesting no interaction between visual and haptic representations. Error bars indicate standard error of the mean across participants, and the plot at 360° is a replica of that at 0°.

**Figure 6:**
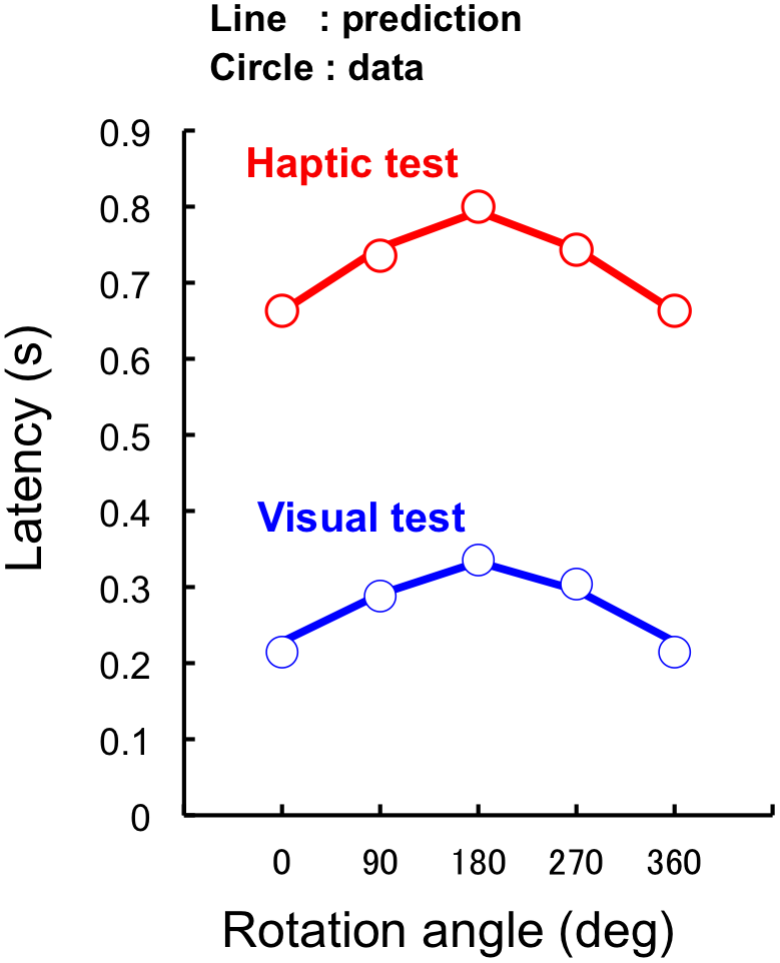
Predicted response latency in consistent trials using latency distribution data obtained in inconsistent trials, assuming random selection from independent representation processes for visual and haptic movements. Selection probability, assumed to be the same for all participants, was determined for the best prediction in terms of least square error.

The learning phase had both consistent and inconsistent trials. Visual and haptic movements were the same in consistent trials and different in inconsistent trials. Three possible inconsistent stimuli corresponded to one consistent stimulus (Fig. 4). To realize similar conditions between consistent and inconsistent trials, we used inconsistent movements symmetrical along either the axis of first stroke or the perpendicular axis. To clearly differentiate between the two modalities in the inconsistent stimulus, we did not use angles between the first and second strokes close to 0°, 90°, 180°, or 270° (excluding the range of −22.5° to +22.5°). In order to equate the numbers of consistent and inconsistent trials, we used the consistent stimulus three times more often than each of the inconsistent stimuli. Test modality was fixed in a session and the learned stimuli to be recalled were chosen randomly for each trial. Two types of sessions differed in test modality (visual test, VV/HV, or haptic test, VH/HH), and both consistent and inconsistent trials were mixed in a session.

For each session, we conducted 192 trials (4 rotations × 4 first stroke directions in the learning phase × 6 consistent/inconsistent combinations [3 inconsistent trials and 3 consistent trials] × 2 types of retrieval stimuli) and randomly divided the trials into two blocks such that a participant ran one block of 96 trials in one day.

#### Statistical tests

We used within-subjects ANOVA to test the effect of rotation angle on latency under various conditions. To compare latencies between data with different rotation angles, we performed *t*-tests with Holm's correction for multiple comparisons.

## Author Contributions

S.S. and T.Y. designed the experiment; T.Y. and K.M. built the experimental setup; T.Y. performed experiments; S.S., T.Y., K.M. and I.K. wrote the manuscript.

## Supplementary Material

Supplementary Information Supplementary information
