# Supplementary material for: Rotation-independent representations for haptic movements
Source: Sci Rep. 2013 Sep 5;3:2595. doi: 10.1038/srep02595 (PMC3763250; doi:10.1038/srep02595)
Supplement: Supplementary Information [file srep02595-s1.doc]

**Supplementary information**

Rotation-independent representations for haptic movements

Satoshi Shioiri*, Takanori Yamazaki, Kazumichi Matsumiya, and Ichiro Kuriki

Research Institute of Electrical Communication

Tohoku University

*Correspondence to shioiri@riec.tohoku.ac.jp


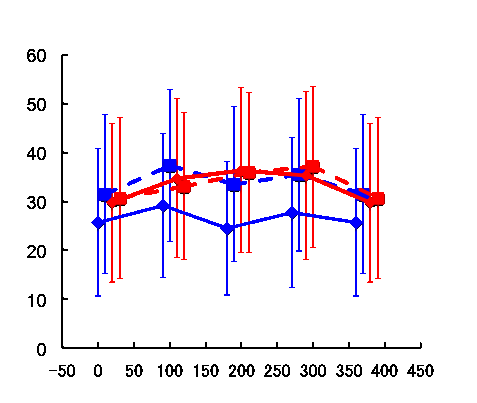


Supplementary Fig. 1

Response accuracy in terms of angle error, that is, the difference in direction between the response goal and the correct location, was calculated for each trial. Average angle error is shown as a function of rotation angle. Each curve corresponds to one of the learning/test combinations. Data points are shifted horizontally for clarity. No systematic difference was found across rotation angles for all conditions although error was smaller in VV trials than in the others. Error bars represent the standard error of the mean and the data points in a condition are plotted within the error bars.


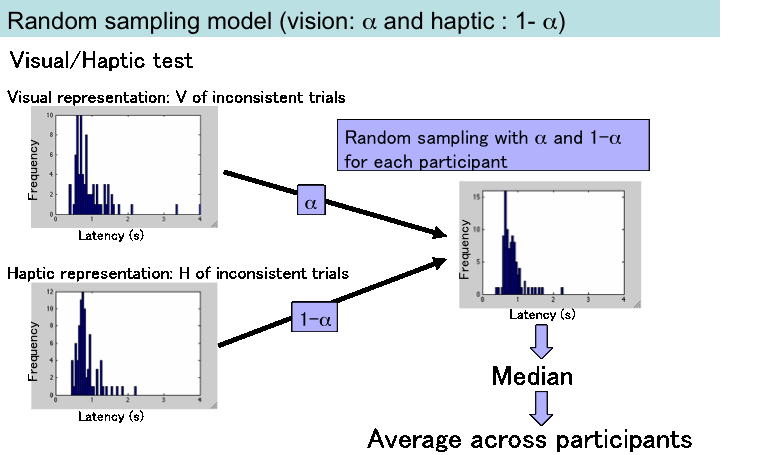


Supplementary Fig. 2

The latency distribution of consistent trials was predicted from the latency distributions of inconsistent trials. With a certain probability, latency data were randomly selected 2100 times (probability of random selection, α, was varied at steps of 0.05, and 100 samples correspond to 5% of the total samples) from the data pool of either visual or haptic learning inconsistent trials. A median was obtained from the latency distribution for each participant, and the average of the median latency was calculated across participants. This process was repeated 1000 times to obtain an estimate of average latency for each fixed probability. Changing the probability, we calculated the least square error for four rotation angle against actual data to find the probability with the best-fitting result. Fig. 6 compares the experimental results (circles) and predictions (lines).
